# Prevalence of cardiovascular events in a population-based registry of patients with systemic lupus erythematosus

**DOI:** 10.1186/s13075-024-03395-6

**Published:** 2024-09-14

**Authors:** Daniel P. Joyce, Jeffrey S. Berger, Allison Guttmann, Ghadeer Hasan, Jill P. Buyon, H. Michael Belmont, Jane Salmon, Anca Askanase, Joan Bathon, Laura Geraldino-Pardilla, Yousaf Ali, Ellen M. Ginzler, Chaim Putterman, Caroline Gordon, Charles G. Helmick, Kamil E. Barbour, Heather T. Gold, Hilary Parton, Peter M. Izmirly

**Affiliations:** 1 New York University Grossman School of Medicine, New York, NY USA; 2 Institute for Rheumatic & Autoimmune Diseases, Atlantic Medical Group Rheumatology, Overlook Medical Center, Atlantic Health System, Summit, Morristown, NJ, NJ USA; 3 Optum Medical Care, North Arlington, NJ USA; 4 Hospital for Special Surgery, New York, NY USA; 5 Columbia University Medical Center, New York, NY USA; 6 Icahn School of Medicine at Mount Sinai, New York, NY USA; 7 SUNY Downstate Health Sciences University, Brooklyn, NY USA; 8 Azrieli Faculty of Medicine, Zefat, Israel; 9 Rheumatology Research Group, Institute of Inflammation and Ageing, University of Birmingham, Birmingham, UK; 10 Independent Researcher, Atlanta, GA USA; 11 Centers for Disease Control and Prevention, Atlanta, GA USA; 12 New York City Department of Health and Mental Hygiene, New York, NY USA

**Keywords:** Systemic lupus erythematosus, Cardiovascular disease, Myocardial infarction, Cerebrovascular accident, Epidemiology

## Abstract

**Background:**

The Manhattan Lupus Surveillance Program (MLSP), a population-based retrospective registry of patients with systemic lupus erythematosus (SLE), was used to investigate the prevalence of cardiovascular disease events (CVE) and compare rates among sex, age and race/ethnicity to population-based controls.

**Methods:**

Patients with prevalent SLE in 2007 aged ≥ 20 years in the MLSP were included. CVE required documentation of a myocardial infarction or cerebrovascular accident. We calculated crude risk ratios and adjusted risk ratios (ARR) controlling for sex, age group, race and ethnicity, and years since diagnosis. Data from the 2009–2010 National Health and Nutrition Examination Survey (NHANES) and the 2013–2014 NYC Health and Nutrition Examination Survey (NYC HANES) were used to calculate expected CVE prevalence by multiplying NHANES and NYC HANES estimates by strata-specific counts of patients with SLE. Crude prevalence ratios (PRs) using national and NYC estimates and age standardized prevalence ratios (ASPRs) using national estimates were calculated.

**Results:**

CVE occurred in 13.9% of 1,285 MLSP patients with SLE, and risk was increased among men (ARR:1.7, 95%CI:1.2–2.5) and older adults (age > 60 ARR:2.5, 95%CI:1.7–3.8). Compared with non-Hispanic Asian patients, CVE risk was elevated among Hispanic/Latino (ARR:3.1, 95%CI:1.4-7.0) and non-Hispanic Black (ARR:3.5, 95%CI1.6-7.9) patients as well as those identified as non-Hispanic and in another or multiple racial groups (ARR:4.2, 95%CI:1.1–15.8). Overall, CVE prevalence was higher among patients with SLE than nationally (ASPR:3.1, 95%CI:3.0-3.1) but did not differ by sex. Compared with national race and ethnicity-stratified estimates, CVE among patients with SLE was highest among Hispanics/Latinos (ASPR:4.3, 95%CI:4.2–4.4). CVE was also elevated among SLE registry patients compared with all NYC residents. Comparisons with age-stratified national estimates revealed PRs of 6.4 (95%CI:6.2–6.5) among patients aged 20–49 years and 2.2 (95%CI:2.1–2.2) among those ≥ 50 years. Male (11.3, 95%CI:10.5–12.1), Hispanic/Latino (10.9, 95%CI:10.5–11.4) and non-Hispanic Black (6.2, 95%CI:6.0-6.4) SLE patients aged 20–49 had the highest CVE prevalence ratios.

**Conclusions:**

These population-based estimates of CVE in a diverse registry of patients with SLE revealed increased rates among younger male, Hispanic/Latino and non-Hispanic Black patients. These findings reinforce the need to appropriately screen for CVD among all SLE patients but particularly among these high-risk patients.

## Background

Systemic lupus erythematosus (SLE) is an autoimmune disease responsible for significantly increased mortality with multi-system organ involvement [1, 2]. Cardiovascular disease (CVD) is a leading cause of death in patients with SLE [1, 3–5], and SLE itself has been identified as an independent risk factor for cardiovascular disease events (CVE) [6, 7]. Although heart disease is the most common cause of death in the general United States population [8], the risks are particularly amplified in patients with SLE, with estimates of 2 to 4 times higher risk [5, 7, 9, 10], including events occurring at younger ages and with increased event mortality [6, 11]. The pathophysiology of this phenomenon has yet to be fully characterized but is largely attributed to earlier atherosclerosis due to increased inflammation in SLE [12].

There is a notably increased burden of SLE on minority populations, including higher disease prevalence and worse mortality outcomes [5, 13–15]. While some studies have compared the CVD rates among racial and ethnic groups with SLE [5, 16–21], there are fewer studies on Hispanic/Latino and Asian American populations or how CVD rates among those with SLE compare with the general population by race or ethnicity. Recently a study from the California Lupus Surveillance Program (CLSP) explored causes of death among patients with SLE and showed that CVD was the leading cause of death and was approximately four and six times higher for Asian and Hispanic/Latino individuals with SLE, respectively, compared with the general population [5]. In this study we leveraged the Manhattan Lupus Surveillance Program (MLSP), which is a Centers for Disease Control and Prevention (CDC)-funded, retrospective, population-based muti-racial and ethnic registry of patients with SLE, to investigate the prevalence of CVE among patients with SLE with the goal of reinforcing appropriate screening and providing clinical guidance.

## Methods

Case-finding methods for the MLSP have been previously described [2]. In short, this registry was created through a HIPAA-exempt health surveillance collaboration between the CDC, the New York University (NYU) Grossman School of Medicine, and the New York City Department of Health and Mental Hygiene (NYC DOHMH). No patients were contacted for this registry, and the MLSP did not require institutional review board (IRB) approval at CDC, NYC DOHMH, or the NYU Grossman School of Medicine, although secondary analyses reported herein were approved by the NYC DOHMH IRB (NYC DOHMH IRB no. 16–147).

The MLSP surveillance period was from 1 January 2007 through 31 December 2009 in New York County (Manhattan), and the program used case-finding sources including rheumatology practices, hospitals, and hospitalization discharge and death registry databases [2]. Sources were queried retrospectively to identify patients who lived in Manhattan with International Classification of Disease Ninth Revision Clinical Modification (ICD-9-CM) billing codes related to SLE. Overall, 90.5% of hospitals and 75.8% of rheumatologists’ practices were included in the MLSP and, for patients residing in Manhattan with one of the ICD-9-CM codes, charts were abstracted by trained abstractors with medical degrees who underwent extensive training and routine quality assurance. Data collected included diagnoses and other elements that were part of the three sets of classification criteria for SLE as previously described [2, 22]. In addition, CVE were captured if documentation (i.e., physician’s documentation, supportive tests, but not ICD-9-CM codes) existed confirming a myocardial infarction (MI) or cerebrovascular accident (CVA), and evidence of either was considered a CVE. If there was no documentation of a CVE in the chart it was considered negative.

Patients with SLE aged 20 years and older residing in Manhattan in 2007 who met one of the sets of SLE classification criteria (1997 ACR [23, 24], SLICC [25], or EULAR/ACR [26]) were included in this study as previously described [22, 27]. We combined information on race and ethnicity of patients abstracted from their medical records into five mutually exclusive categories: Hispanic/Latino (regardless of evidence of another race), non-Hispanic White, non-Hispanic Black, non-Hispanic Asian, and non-Hispanic other (including multiple races). Crude risk ratios were calculated by sex, age group, and race and ethnicity, and adjusted risk ratios (ARR) were calculated controlling for sex, age group, race and ethnicity, and number of years since SLE diagnosis. We used interview data from the 2009–2010 National Health and Nutrition Examination Survey (NHANES) and the 2013–2014 NYC Health and Nutrition Examination Survey (NYC HANES) to determine the prevalence of heart attack or stroke nationally and in NYC, respectively [28, 29]. Prevalence estimates for those identified as non-Hispanic Asian and non-Hispanic other were not included, as they were either not available from the data source (NHANES) or estimates were unreliable (NYC HANES). We calculated the expected prevalence of heart attack or stroke by multiplying national and NYC-specific prevalence estimates by strata-specific counts of patients with SLE in the MLSP. We then calculated unadjusted prevalence ratios overall, by sex, and race and ethnicity using both national and NYC estimates. Age-standardized prevalence ratios (ASPR) were calculated by determining prevalence of heart attack or stroke among three age groups (20–39, 40–59, 60+) using the national estimates, multiplying age-stratified national prevalence estimates by the number of MLSP patients to determine the number of expected cases of heart attack or stroke, and then taking the ratio of total cases observed to total cases expected. ASPRs using the NYC estimates were not calculated as prevalence estimates stratified by age group were unreliable. Utilizing the same methodology to calculate prevalence ratios, further analyses by race and ethnicity and sex were made to determine age effects in these SLE subgroups compared to NHANES controls. Given the smaller subgroup sizes, two age groups (20–49 and 50+) were used in a direct comparison. All analyses were completed using SAS version 9.4 (SAS Institute Inc., Cary, NC, USA).

## Results

### Demographics of Manhattan patients with SLE with Cardiovascular events

CVE (MI or CVA) were observed in 179 out of 1,285 (13.9%) of criteria-defined MLSP patients with SLE, Table 1. 41 patients were excluded due to missing racial and ethnic information. CVE prevalence was higher among male patients than female patients (21.3% vs. 13.3%); the ARR of CVE among male compared with female patients with SLE was 1.7 (95% CI: 1.2–2.5), Table 1. CVE prevalence increased with age (8.1% among those aged 20–39 years; 13.9% among those aged 40–59 years; and 26.1% among those aged 60 years or older, Table 1). Compared with MLSP patients with SLE aged 20–39, those aged 40–59 had an ARR for a CVE of 1.4 (95% CI: 1.0–2.0), while those aged 60 or older had an ARR of 2.5 (95% CI: 1.7–3.8), Table 1. CVE occurred among 12.4% of non-Hispanic White patients, 17.5% of non-Hispanic Black patients, 16.6% of Hispanic/Latino patients, 4.3% of non-Hispanic Asian patients, and 9.3% of those identified as non-Hispanic other. Compared with non-Hispanic Asian patients, non-Hispanic White patients had an ARR of 1.9 (95% CI: 0.9–4.4); Hispanic/Latino patients, 3.1 (95% CI: 1.4–7.0); non-Hispanic Black patients, 3.5 (95% CI: 1.6–7.9); and non-Hispanic other, 4.2 (95% CI: 1.1–15.8), Table 1.

**Table 1:** Demographics and risk ratios for SLE cardiovascular events - Manhattan Lupus Surveillance Program, 2007

	Any CVE	All patients with SLE	Percent	Crude risk ratio	Adjusted risk ratio* (95% CI)
Total	179	1285	13.9		
Male	23	108	21.3	1.6	1.7 (1.2–2.5)
Female	156	1177	13.3	(ref)	(ref)
Age group					
20–39	41	504	8.1	(ref)	(ref)
40–59	75	540	13.9	1.7	1.4 (1.0–2.0)
60+	63	241	26.1	3.2	2.5 (1.7–3.8)
Race and Ethnicity					
Non-Hispanic White	48	388	12.4	2.8	1.9 (0.9–4.4)
Non-Hispanic Black	58	331	17.5	4.0	3.5 (1.6–7.9)
Hispanic/Latino	62	374	16.6	3.8	3.1 (1.4–7.0)
Non-Hispanic Asian	6	138	4.3	(ref)	(ref)
Non-Hispanic Other	5	54	9.3	2.1	4.2 (1.1–15.8)

CVE = cardiovascular disease events.

Outcomes include myocardial infarction and cerebrovascular disease.

Patients include residents of Manhattan in 2007 with a new or existing diagnosis of SLE by ACR, EULAR, or SLICC criteria who are aged 20 or older.

*Adjusted risk ratios were calculated from a Poisson model which incorporated sex, age group, race and ethnicity, and years since SLE diagnosis. 41 patients were excluded due to missing racial and ethnic information.

### Prevalence Ratios of CVE in SLE compared with general populations

**Fig. 1:**
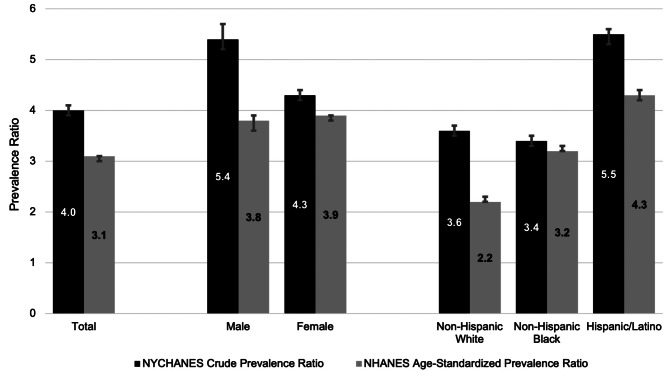
Crude and age-standardized CVE prevalence ratios for MLSP patients with SLE, overall and by subgroup. Figure 1 footnote 1: NYCHANES data from 2013–2014, NHANES data from 2009–2010 MLSP, 2007 among Manhattan Residents. Figure 1 footnote 2: Prevalence estimates for those identified as non-Hispanic Asian and non-Hispanic other were not included, as they were either not available from the data source (NHANES) or estimates were unreliable (NYC HANES). However, they were not excluded from total or sex-specific ratios presented. Age-standardized prevalence ratios (ASPR) were calculated based on prevalence of heart attack or stroke among three age groups (20–39, 40–59, 60+)

Compared with a 2009–2010 national estimate from NHANES, the ASPR of CVE among all MLSP patients with SLE was 3.1 (95% CI: 3.0–3.1) overall and 3.8 (95% CI: 3.6–3.9) for male patients and 3.9 (95% CI: 3.8–3.9) for female patients, Fig. 1. ASPRs for CVE among MLSP patients with SLE by race and ethnicity were 2.2 (95% CI: 2.2–2.3) for non-Hispanic White patients, 3.2 (95% CI: 3.2–3.3) for non-Hispanic Black patients, and 4.3 (95% CI: 4.2–4.4) for Hispanic/Latino patients, Fig. 1.

When compared with estimates of CVE prevalence in New York City using 2013–2014 NYCHANES data, the unadjusted prevalence ratio of CVE among MLSP patients with SLE was 4.0 (95% CI: 3.9–4.1). Male and female patients with SLE had unadjusted CVE prevalence ratios of 5.4 (95% CI: 5.2–5.7) and 4.3 (95% CI: 4.2–4.4), respectively, Fig. 1. Non-Hispanic White MLSP patients with SLE had an unadjusted CVE prevalence ratio of 3.6 (95% CI: 3.5–3.7), while non-Hispanic Black and Hispanic/Latino patients had unadjusted CVE prevalence ratios of 3.4 (95% CI: 3.3–3.5) and 5.5 (95% CI: 5.3–5.6), respectively, compared with their respective race and ethnicity-matched estimates for NYC.

**Fig. 2:**
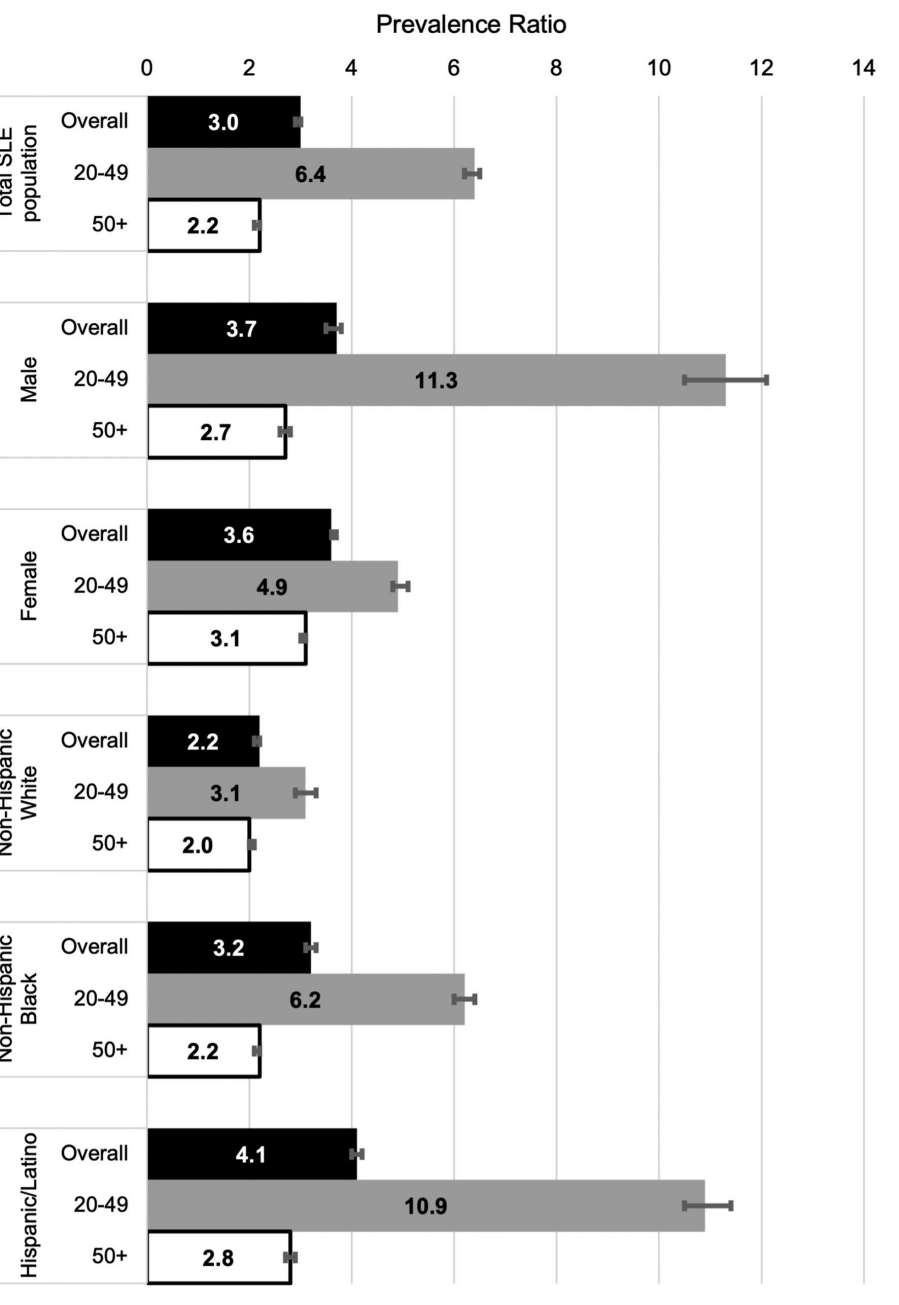
CVE prevalence ratios among MLSP patients with SLE by age-group, sex, and race and ethnicity. Figure 2 footnote: Adjusted prevalence ratios comparing MLSP data from 2007 among Manhattan Residents to NHANES data from 2009–2010. Age-standardized prevalence ratios (ASPR) were calculated based on prevalence of heart attack or stroke among (20–49 and 50+)

We also calculated CVE prevalence ratios by age group among MLSP patients with SLE compared with national estimates, Fig. 2. The SLE CVE prevalence ratio was 6.4 (95% CI: 6.2–6.5) among patients aged 20–49 and 2.2 (95% CI: 2.1–2.2) among those aged 50 and older. Among male MLSP patients with SLE, the CVE prevalence ratios were 11.3 (95% CI: 10.5–12.1) for those aged 20–49 and 2.7 (95% CI: 2.6–2.8) for those aged 50 and older compared with the national estimate. Among female MLSP patients with SLE, CVE prevalence ratios were 4.9 (95% CI: 4.8–5.1) for those aged 20–49 and 3.1 (95% CI: 3.0–3.1) for those aged 50 and older compared with national estimates. For non-Hispanic White MLSP patients with SLE, those aged 20–49 had a CVE prevalence ratio of 3.1 (95% CI: 2.9–3.3) compared with the non-Hispanic White population nationally, whereas those aged 50 and older had a prevalence ratio of 2.0 (95% CI: 2.0–2.1) compared with the national estimate. Among non-Hispanic Black MLSP patients with SLE, the CVE prevalence ratios among those aged 20–49 and those 50 and older were 6.2 (95% CI: 6.0–6.4) and 2.2 (95% CI: 2.1–2.2), respectively, compared with national estimates. And finally, among Hispanic/Latino MLSP patients with SLE, those aged 20–49 had a CVE prevalence ratio of 10.9 (95% CI: 10.5–11.4) compared with the national estimate, while those aged 50 and older had an adjusted prevalence ratio of 2.8 (95% CI: 2.7–2.9) compared with the national estimate, Fig. 2.

## Discussion

Since the seminal work associating SLE and CVD [4, 11], numerous studies have been conducted to further characterize this relationship. However, data to fully characterize rates by race and ethnicity, particularly among Hispanic/Latino and Asian American populations, have been scant. Leveraging the MLSP has provided data that corroborated and expanded upon the existing association between CVD and SLE. Our findings in a heterogeneous population-based cohort support the now well-characterized association that patients with SLE have higher rates of CVE than the general population. CVE occurred in nearly 14% of MLSP patients with SLE with male sex, non-Hispanic Black race, Hispanic/Latino ethnicity, and age being risk factors. Compared with national and NYC data on the general population, patients with SLE in the MLSP had a higher risk of CVE with the highest ratios seen in Hispanic/Latino patients with SLE and male patients with SLE. Comparisons to national estimates by age group revealed that younger SLE patients have an increased risk for CVE, with the highest risk seen in SLE male patients and Hispanic/Latino patients with SLE younger than 50.

Through a wide range of population-based cohorts, increased CVE and CVD mortality have been identified among patients with SLE, particularly at younger ages [5, 9, 20, 30–34]. Additional research shows increased atherosclerosis among patients with SLE via carotid ultrasound measurements and cardiac calcium score measurements [35–37]. The data presented herein of an increased SLE rate of CVE of 3.1 are similar to findings from the 2009 Nurse’s Health Study [9], showing a risk ratio of 2.8, a meta-analysis of MI and stroke risk in SLE that showed risk ratios of 3.0 and 2.1, respectively, and a recent nationwide study from the United Kingdom showing a risk ratio of 2.8 [38].

Factoring in race and ethnicity provides several meaningful takeaways. When comparing MLSP patients with SLE with their respective racial and ethnic categories nationally, Hispanic/Latino patients with SLE had the highest CVE ASPR of 4.3, followed by non-Hispanic Black patients with 3.2. Demonstrating the higher rates of CVD among non-Hispanic Black and Hispanic/Latino patients with SLE in the MLSP echoes the literature indicating the increased disease burden on minority patients [13, 14]. Race- and ethnicity-stratified estimates of CVD among patients with SLE have demonstrated disparities among Black patients with SLE compared with White patients with SLE. In 2022, the Georgia Lupus Registry performed a similar characterization of CVE rates between Black and non-Black patients with SLE which demonstrated 19-fold higher rates of CVE in Black patients with SLE compared with non-Black patients with SLE in the first 12 years of surveillance [17]. Studies of the LUMINA (Lupus in Minorities: Nature vs. Nurture), Medicaid, and Chicago/Pittsburgh populations have similarly demonstrated higher rates of CVE or atherosclerosis among Black patients compared with White patients [16, 19, 21], though not all reached statistical significance. Our findings also demonstrate increased CVE rates among non-Hispanic Black MLSP patients with SLE compared with a general non-Hispanic Black population, a direct comparison which has yet to be fully characterized. The finding that non-Hispanic Black MLSP patients with SLE have a higher ASPR than non-Hispanic White MLSP patients with SLE suggests that disparities among SLE patients may be worse than the disparities seen among the general population.

Recently, data from the California Lupus Surveillance Program (CLSP) showed that CVD was the leading cause of death among patients with SLE and that CVD death was nearly 6 times higher among Hispanic individuals with SLE compared with the general population [5]. Previous data on CVD among Hispanic SLE populations comes primarily from the LUMINA and Medicaid studies [16, 21]. The LUMINA study found no statistical association between Hispanic ethnicity and increased CVD risk compared with non-Hispanic White patients with SLE, though numbers trended towards lower rates of vascular events among Hispanic patients [21]. The Medicaid study demonstrated a decreased rate of CVD among Hispanic SLE populations compared with White SLE populations with a significant risk ratio of 0.84 (95% CI: 0.79–0.90) [16]. Our study showed increased rates of CVD in this ethnic group corroborating findings from the CLSP mortality study [5].

Stratification by sex demonstrates that among patients with SLE included in the MLSP, CVE occurred at significantly higher rates among males compared with females, echoing rates of CVE in the general population. However, similarly increased ASPRs for CVE were observed among male and female MLSP patients with SLE when compared with the national population. The increase in the unadjusted PRs for CVE was slightly higher in males than females when compared with the NYC population. A meta-analysis of the association between atherosclerotic CVD and SLE identified that traditional risk factors such as male sex also represent higher risks in SLE [39], while the Swiss SLE cohort also demonstrated that male patients with SLE were found to have worse cardiovascular and renal disease severity [40].

The number of CVE events among MLSP cases of SLE increased with older age; however, when compared with the general national population, the increased relative risk appears to be greater among those aged 20–49 than those aged 50 and older. This result has been shown in many age-focused analyses, including a 1997 study, which demonstrated a greater than 50-fold increase in coronary heart disease incidence among younger women, aged 35–44 [11], and many population-based studies demonstrating increased CVD risk at younger ages [20, 34, 38, 41–43]. Presumably, this is due to other CVD risk factors “catching up” to the risk that patients with SLE incur as they age, while younger patients may have fewer overall contributing factors than the general population and factors intrinsic to SLE become the major drivers. ASPRs for CVE outcomes were particularly elevated for MLSP patients with SLE aged 20–49 in three demographic subgroups: male (11.3), Hispanic/Latino (10.9), and non-Hispanic Black patients (6.2). These patients comprise groups (males and minorities with SLE) which often have worse outcomes [13, 14, 44] that may in part be due to delays or lapses in early diagnosis and care or more significant environmental impacts.

Although SLE has been shown to have comparable or even higher CVD risk compared with other systemic diseases such as type 2 diabetes and rheumatoid arthritis [42, 45–47], most traditional cardiovascular risk score calculators, such as the Framingham Risk Score and the ACC’s ASCVD risk score calculator, factor diabetes into the calculation, but not SLE [48, 49]. Recent CVD risk score calculators such as the QRISK3 [50] have been inclusive of SLE and its heightened CVD prevalence but are not widely used. A recent study in patients with SLE showed QRISK3 demonstrated better performance in predicting risk of cardiovascular disease in a SLE cohort compared with traditional calculators [51]. An SLE-specific calculator using machine learning has also been found to be more sensitive for predicting CVE in SLE than traditional calculators [52].

The present study does have several limitations. Given the surveillance nature of the MLSP, data on traditional CVD risk factors such history of smoking, diabetes and hypertension, were not collected. With a large cohort such as this, the potential for a confounding cardiovascular risk factor to impact differences across groups is reduced but not fully eliminated. NHANES and NYC HANES estimates likely include patients with SLE which may have reduced the CVE risk ratio. Additionally, relevant NYC HANES data was only available for 2013–2014, so we assumed that the underlying prevalence of CVE in NYC did not change between 2007 and 2013–2014. In addition, SLE-associated risk factors such as steroid use and renal disease which can confound CVD were not analyzed [11, 37, 53]. The lack of reliable comparison data for non-Hispanic Asian patients and those identified as being non-Hispanic and a member of another or multiple racial groups also limits complete assessments of racial and ethnic outcomes in CVD.

We also recognize the limitations of reported and aggregated race and ethnicity generally, which may poorly capture disparities, particularly amongst Hispanic/Latino, non-Hispanic Asian, and other racial and ethnic subgroups [54]. Creating mutually exclusive racial and ethnic subgroups generates a necessity to divide individuals who may hold multiple or complex racial and ethnic identities into socially determined strata, which typically serve as a proxy for other factors of social determinants of health. Utilizing the medical records’ reported race and ethnicity also has the potential for an inaccurate coding of how a patient truly identifies. However, such error is likely present in any analysis of race and ethnicity using medical records.

An additional limitation is the underlying potential for miscoding or misidentification of CVE due to variation in data capture by medical records and the NYC HANES and NHANES questionnaires. However, such errors are unlikely to be significantly misrepresented in comparison to any other EMR coding technique; thus, the underlying uncertainty is likely present in any large population-based study such as this one. Further, with regard to outcomes, we combined MI and CVA to provide greater power over analyzing each outcome individually, which may simplify findings in the present study.

A final limitation is the time that has elapsed since data collection in 2007. Although ideally outcomes for SLE, CVD, and health disparities have improved since 2007, the differences among population subgroups still allow for valuable comparison in 2024 and are in line with previous published work. Furthermore, the dataset provides a unique ability to categorize by subgroups within a whole population-based cohort.

The MLSP has notable strengths compared with past studies. The data represent a large sex, racially and ethnically diverse SLE patient population in Manhattan, providing significantly more generalizable conclusions and greater numbers for comparison of demographic groups that had not been well-characterized previously. The data collection procedure also has significant strengths over many in that SLE diagnoses are verified by the rheumatology criteria for SLE, and designations of CVE were captured from the patients’ medical records and coded by trained medical abstractors who had medical degrees, which offers a more accurate way of categorizing validated SLE and the outcomes that come from it [2, 22]. In addition to the sex, racial and ethnic diversity, the dataset al.lows comparison of demographic groups and includes a patient population across a geographic region with more rheumatologists and health care systems than other population studies. Further, the population-based nature of the MLSP allowed for richer prevalence comparisons with a high-quality, standardized national dataset and an NYC-specific dataset (NHANES and NYC HANES).

## Conclusions

In summary, the MLSP population-based estimates of CVE in patients with SLE revealed higher risk ratios compared with the general national and local populations. Rates were strikingly increased in younger male, Hispanic/Latino and non-Hispanic Black patients with SLE. These findings should reinforce the need to appropriately screen for CVD in all patients with SLE but particularly in these high-risk patients. Given the increased risk of CVD associated with SLE, adjustments should be made to current risk calculators for SLE, and utilization of an SLE-inclusive risk calculator should be prioritized.

## Data Availability

This study used data from a surveillance initiative which are housed at the NYC DOHMH. Requests to access these datasets should be directed to hparton@health.nyc.gov.

## Abbreviations

ACC: American College of Cardiology
ACR: American College of Rheumatology
ARR: Adjusted Risk Ratio
ASCVD: Atherosclerotic Cardiovascular Disease
ASPR: Age Standardized Prevalence Ratio
CDC: Centers for Disease Control and Prevention
CLSP: California Lupus Surveillance Project
CVA: Cerebrovascular Accident
CVD: Cardiovascular Disease
CVE: Cardiovascular Disease Events
EULAR: European Alliance of Associations for Rheumatology
ICD: 9–CM–International Classification of Disease–9th Revision–Clinical Modification
LUMINA: Lupus in Minorities: Nature vs. Nurture
MI: Myocardial infarction
MLSP: Manhattan Lupus Surveillance Project
NHANES: National Health and Nutrition Examination Survey
NYCDOHMH: New York City Department of Health and Mental Hygiene
NYC HANES: New York City Health and Nutrition Examination Survey
PR: Prevalence Ratio
SLE: Systemic Lupus Erythematosus
SLICC: Systemic Lupus Erythematosus Collaborating Clinics
95%CI: 95% Confidence Interval
